# Dataset of human interventions as anthropogenic perturbations on the Caribbean coast of Colombia

**DOI:** 10.1016/j.dib.2020.105847

**Published:** 2020-06-12

**Authors:** C.M. Botero, C.I. Pereira, C.B. Milanes, E. Pranzini

**Affiliations:** a School of Law, Universidad Sergio Arboleda, Colombia; b Coastal Systems Research Group, Playas Corporacion Ltd, Colombia; c Civil and Environmental Department, Universidad de la Costa, Colombia; d Earth Sciences Department, Universita di Firenze, Italy

**Keywords:** Environmental impact assessment, Coastal management, Blue economy, Caribbean Sea, Google Earth imagery

## Abstract

Human interventions on coastal areas are always causing environmental impact; however, most of the times inventories of those interventions are possibly not well structured, and surely without a specific standard. The raw data presented shows an exhaustive and systematic revision of satellite images on 1700 km of the Caribbean coast of Colombia, where 2743 human interventions were identified. These interventions are classified in 38 categories in order to assess their environmental impact at a regional scale. The filtered data shows the environmental impact obtained for each category and the values allotted to each of the four parameters used for this evaluation. Moreover, the data is filtered for each of the five environmental coastal units in which the Caribbean coast of Colombia is divided by national regulations. Finally, the filtered and processed data shows the analysis done to obtain the graphical results of a previously paper (An evaluation of human interventions in the anthropogenically disturbed Caribbean Coast of Colombia [1]). Therefore, this dataset comprises three spreadsheets (xlsx) and two geographical files (kmz), which are ready to be used for any researcher, decision maker, land planner or practitioner interested in making further analysis on environmental impact assessment in coastal areas. Additionally, the dataset is carefully organised for educational exercises in such a manner that professors or lecturers can repeat the same steps in this study area or in their own, from the inventory to the final results.

Specifications table

**Table:** 

Subject	Environmental Engineering
Specific subject area	Environmental impact on coastal areas, human interventions
Type of data	Tables, Geographical locations
How data were acquired	Digital survey of approximately 1700 km of coastline, through available imagery in Google Earth Pro by June 2017
Data format	Raw, Filtered, Analysed
Parameters for data collection	Data is structured from the position marks done in Google Earth, where human interventions were identified. The first parameter was the human intervention, in which each datum has two kinds of information: a. Type of intervention; b. Metadata about the image shown in Google Earth. The second parameter is the simplified environmental impact assessment obtained from the evaluation of four attributes and the interventions account within the 38 categories of human interventions.
Description of data collection	During three months of GIS-Lab work, every position mark of human interventions was registered on a spreadsheet, covering more than 1700 km of coastline.
Data source location	Continental Caribbean coast of Colombia: eight geographical departments (Choco, Antioquia, Córdoba, Sucre, Bolivar, Atlantico, Magdalena, Guajira)
Data accessibility	With the article
Related research article	Pereira, C.I., Madrid, D., Correa, I.D., Pranzini, E., Botero, C.M., An evaluation of human interventions in the anthropogenically disturbed Caribbean Coast of Colombia, Anthropocene 27 (2019) 100,215 (1–11) DOI: https://doi.org/10.1016/j.ancene.2019.100215

## Value of the data

• This dataset of human interventions allows to do several extra and derived analysis of the environmental impact caused on Colombian coastal zones, with emphases on the 1700 km on the continental Caribbean seafront.
• The calculation to obtain the simplified environmental impact assessment is of great interest to researchers and technicians looking for examples of quick and reliable EIA examples.
• This dataset shows step by step how to identify and register human interventions in coastal areas using an open source tool such as Google Earth. It also shows how to process, calculate and graphically represent the environmental impact in a simple way, which could be very useful for professors in environmental and marine sciences.
• The dataset is formed by three spreadsheets, which allow future researchers and practitioners to repeat the same process in three levels of complexity: raw data for inventory of human interventions, filter and process data for calculations of environmental impact and analysed data for statistical and graphical representations.
• The dataset can be used as a baseline for long-term monitoring of the human interventions on the Caribbean coast of Colombia and their environmental impact on coastal and marine ecosystems.

## 1 Data description

The dataset contains five files: three spreadsheets in MS Excel format (xlsx) and two geographical files in Google Earth format (kmz), which are presented as supplementary material. The first spreadsheet (*DiB_Intervencoast_tables_Raw*) includes the raw data of all 2743 human interventions found on the Caribbean coast of Colombia, and is used to register an inventory of 1700 km of coastline. This raw data file has 40 datasheets in which the first shows the seven categories and 38 types of human interventions used, with their codes, descriptions and quantity of data (Table 1). The second datasheet consolidates all the human interventions identified in the five Environmental Coastal Units (ECU) of the study area, which adds up to 3957 records. The rest of the 38 datasheets show human interventions in each typology, describing the ECU, position mark, geocode in the kmz files, date of the satellite image and the satellite source; the datasheets of each category have the same colour as the one used in the first descriptive datasheet to make their usage easy (Table 1). The differences between the total number of records (3957) and the number of interventions (2743) follow the distinctive geographical representations for the identified interventions. Some interventions were marked as polygons of four vertices (e.g. aquaculture farms, towns, condominiums), others as lines of two vertices (e.g. roads, groins/jetties) and the rest as single points (e.g. hotels, military bases, ports). Therefore, the polygons have four records, corresponding to the four cardinal extreme points (N, E, S, W), and the lines have two records, one for each extreme point.

**Table 1:** Categories, types and description of human interventions in coastal areas and quantity of data for the Caribbean coast of Colombian.

The second spreadsheet (*Intervencoast_tables_filtered.xlsx*) has five datasheets with consolidated, filtered and processed data. The first datasheet includes the frequency of 38 human interventions in each typology per each ECU (Table 2). The rows show the name and code of each type of intervention, the number of interventions in the five ECU and the total interventions in each typology. Additionally, this datasheet shows the simplified environmental impact assessment done to each intervention typology (Table 3). This section has twelve rows that could be classified in three groups: the first three rows show the type of intervention, their frequency of occurrence and their percentage over the total interventions count; the following six rows are the parameters (EXT=extension; INT=intensity; REV=reversibility; PER=persistence) used to calculate the Unitary Environmental Impact (UEI; fifth row) and the proportion in the overall UEI; the final three rows show the Total Environmental Impact (TEI) for each intervention type, which is a function of the UEI and the frequency of occurrence, the proportion in the overall TEI of the study area and the accumulated frequency of TEI values.

**Table 2:** Human interventions in each environmental coastal unit of the Caribbean coast of Colombia.

Coastal intervention		Environmental coastal unit*					Total
		GUAJIRA	VNSMR	MAGDIQUE	SINU	DARIEN	
Low density settlements	AHB	306	83	62	283	237	971
High density settlements	AHA	0	5	10	2	1	18
Palafitical settlements	AHP	0	0	2	0	0	2
Luxury settlements	AHU	0	16	89	33	7	145
Luxury settlement with pier	AHM	0	3	146	39	0	188
Walks and ridges	PYC	0	3	3	1	0	7
Public docks	MUP	9	2	11	17	11	50
Road infrastructure	CAP	3	6	33	12	9	63
Railway infrastructure	VFE	0	1	0	0	0	1
Electrical installations	INE	0	0	0	0	0	0
Pipelines (gas/oil)	POL	0	0	0	0	0	0
Breakwaters	ROM	0	1	37	13	5	56
Inlet navigation channels	EMP	3	0	23	4	0	30
Groins/jetties	CYP	32	42	211	349	104	738
Seawalls	MUR	2	1	9	2	27	41
Beach nourishments	RPL	0	0	0	0	0	0
Water and sewage pipelines	TAD	0	0	0	0	0	0
Land-based military installations	IMI	0	0	0	0	0	0
Naval military installations	INA	0	1	3	0	2	6
Offshore Platforms	PTF	0	0	0	0	0	0
Mining	DMI	0	0	2	0	0	2
Farming and livestock	UAG	2	11	7	25	17	62
Mariculture	GRM	0	0	1	1	0	2
Aquaculture	GRA	6	6	24	21	4	61
Manufacture	MAN	5	4	14	2	2	27
Thermoelectric plants	TYS	0	1	1	0	0	2
Desalination plants	DES	0	0	0	0	0	0
Internal Maritime Transport	NAV	7	10	19	3	11	50
Deep water ports without shelter	PUC	0	2	10	0	0	12
Shallow water ports without shelter	PUG	3	1	11	1	2	18
Bulk ports	PUP	0	0	0	0	0	0
Fishing ports	PUQ	0	0	0	0	0	0
Cruise tourism	MCR	0	0	1	0	0	1
Marinas	MMN	0	1	17	1	0	19
Sun and Beach Tourism	EDF	0	12	39	4	2	57
Nature Tourism	EDN	4	56	9	28	2	99
Sun and beach tourism with pier	EDM	0	0	6	4	0	10
Historic structures	ESH	0	1	4	0	0	5
TOTAL		382	269	804	845	443	2743

* (GUAJIRA: La Guajira peninsula; VNSMR: Northern slope of the Sierra Nevada de Santa Marta; MAGDIQUE: Magdalena Delta and Canal del Dique; SINU: The Sinu Delta and DARIEN: The Darien Gulf).

**Table 3:** Simplified Environmental Impact Assessment of human interventions on the study area ordered by frequency.

Type	freq	% Total freq	Ext	Int	Rev	Per	UEI	% Total UEI	TEI	% Total TEI	ACUM TEI
AHB	971	35.40	1	1	1	2	0.16	1.12	151.72	18.34	0.18
CYP	738	26.90	5	2	2	4	0.41	2.92	299.81	36.25	0.55
AHM	188	6.85	2	4	2	4	0.38	2.70	70.50	8.52	0.63
AHU	145	5.29	1	4	2	4	0.34	2.47	49.84	6.03	0.69
EDN	99	3.61	1	1	1	1	0.13	0.90	12.38	1.50	0.71
CAP	63	2.30	4	4	4	4	0.50	3.60	31.50	3.81	0.74
UAG	62	2.26	4	2	1	2	0.28	2.02	17.44	2.11	0.77
GRA	61	2.22	2	4	2	4	0.38	2.70	22.88	2.77	0.79
EDF	57	2.08	1	4	2	4	0.34	2.47	19.59	2.37	0.82
ROM	56	2.04	5	2	4	4	0.47	3.37	26.25	3.17	0.85
MUP	50	1.82	2	2	2	4	0.31	2.25	15.63	1.89	0.87
NAV	50	1.82	2	2	2	2	0.25	1.80	12.50	1.51	0.88
MUR	41	1.49	6	4	2	4	0.50	3.60	20.50	2.48	0.91
EMP	30	1.09	6	8	4	4	0.69	4.94	20.63	2.49	0.93
MAN	27	0.98	1	2	2	2	0.22	1.57	5.91	0.71	0.94
MMN	19	0.69	4	4	4	4	0.50	3.60	9.50	1.15	0.95
AHA	18	0.66	4	8	4	4	0.63	4.49	11.25	1.36	0.96
PUG	18	0.66	4	4	4	4	0.50	3.60	9.00	1.09	0.98
PUC	12	0.44	4	4	4	4	0.50	3.60	6.00	0.73	0.98
EDM	10	0.36	5	4	2	4	0.47	3.37	4.69	0.57	0.99
PYC	7	0.26	2	2	4	4	0.38	2.70	2.63	0.32	0.99
INA	6	0.22	2	2	2	4	0.31	2.25	1.88	0.23	0.99
ESH	5	0.18	1	1	2	4	0.25	1.80	1.25	0.15	1.00
TYS	2	0.07	1	4	2	1	0.25	1.80	0.50	0.06	1.00
DMI	2	0.07	4	8	4	4	0.63	4.49	1.25	0.15	1.00
GRM	2	0.07	2	4	1	2	0.28	2.02	0.56	0.07	1.00
AHP	2	0.07	2	2	1	4	0.28	2.02	0.56	0.07	1.00
VFE	1	0.04	4	4	4	4	0.50	3.60	0.50	0.06	1.00
MCR	1	0.04	2	4	4	4	0.44	3.15	0.44	0.05	1.00
INE	0	0.00	4	1	1	2	0.25	1.80	0.00	0.00	1.00
POL	0	0.00	4	2	1	2	0.28	2.02	0.00	0.00	1.00
RPL	0	0.00	2	4	2	2	0.31	2.25	0.00	0.00	1.00
TAD	0	0.00	2	4	1	1	0.25	1.80	0.00	0.00	1.00
IMI	0	0.00	2	2	2	2	0.25	1.80	0.00	0.00	1.00
PTF	0	0.00	2	4	1	2	0.28	2.02	0.00	0.00	1.00
DES	0	0.00	1	4	1	1	0.22	1.57	0.00	0.00	1.00
PUP	0	0.00	2	4	4	4	0.44	3.15	0.00	0.00	1.00
PUQ	0	0.00	2	2	4	4	0.38	2.70	0.00	0.00	1.00
TOTAL	2743	1	–	–	–	–	13.91	–	827.06	–	–

**Fig. 1:**
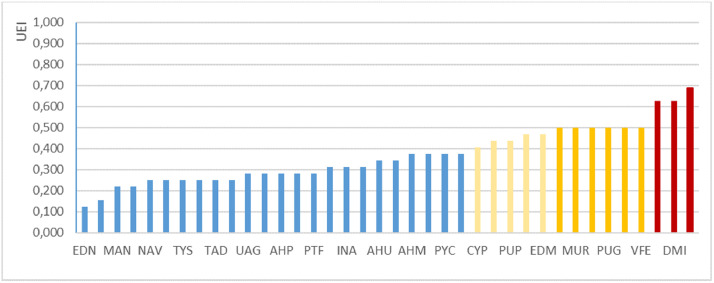
Unitary environmental impact of each human intervention typology. (For interpretation of the references to colour in this figure, the reader is referred to the web version of this article.)

**Fig. 2:**
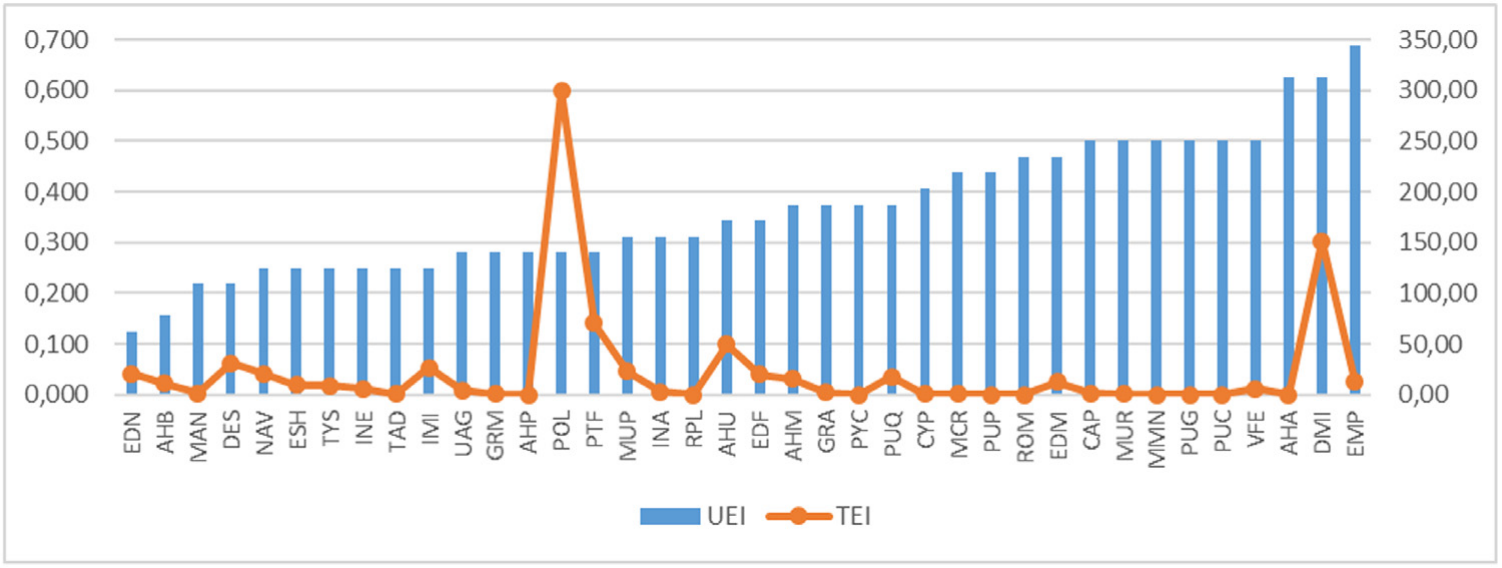
UEI versus TEI in absolute values.

**Fig. 3:**
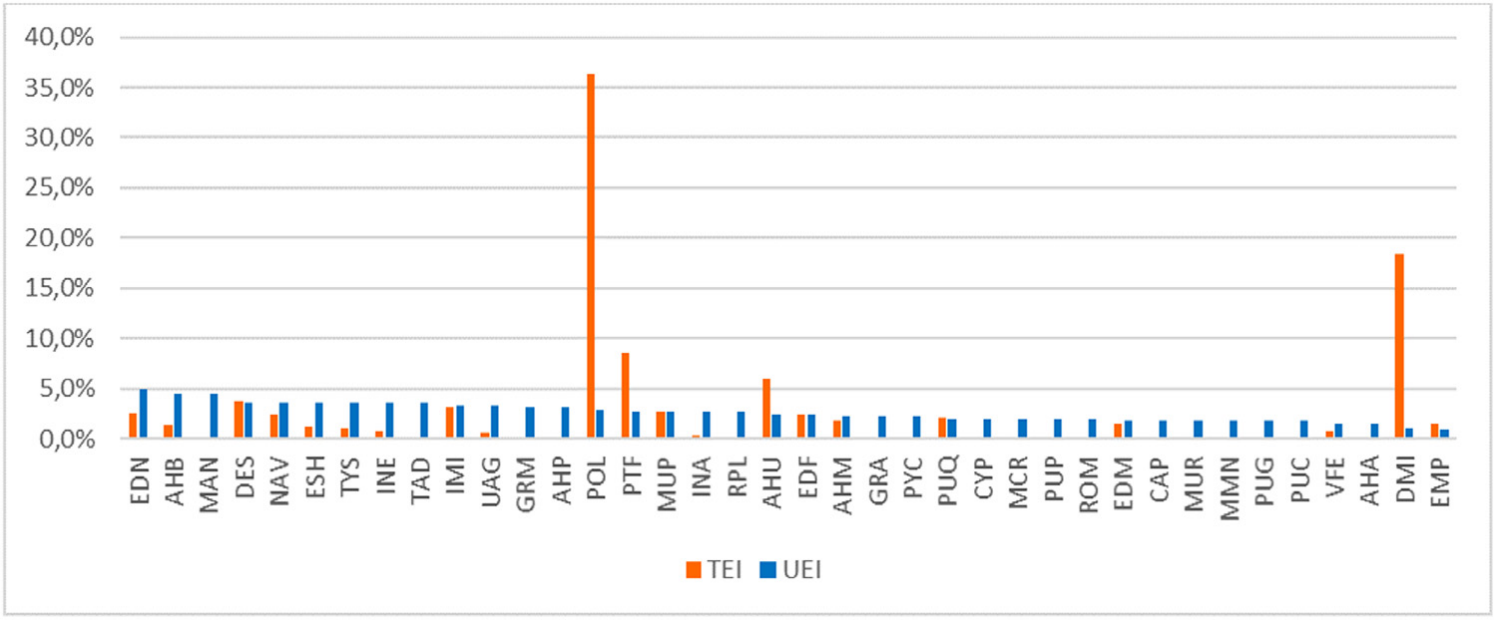
UEI versus TEI in normalised values.

The second datasheet of *Intervencoast_tables_filtered.xlsx* has the filtered data used to graph the main frequency patterns of human interventions on the Caribbean coast of Colombia. Fig. 1 shows the UEI value for each typology, adding a colour for each quartile (Q1 = red; Q2 = Orange; Q3 = Yellow; Q4 = Blue). Fig. 2 shows the comparison between the UEI values versus the TEI values obtained by each typology; because UEI and TEI units have different scales of magnitude, the left side of the Y axis is for UEI and the right side is for TEI. Fig. 3 shows the same comparison, but using normalised values for UEI and TEI in order to allow comparisons in the same order of magnitude.

The third datasheet of *Intervencoast_tables_filtered.xlsx* shows the same data of the first one but filtered to the 29 typologies found in the study area. These filtered data were those used by the article [1], and for the pie graphics shown in the fourth datasheet, which represent the distribution of each typology in each of the five ECU. Moreover, a pie graph with the consolidated data of the five ECU is also included. The last datasheet shows the UEI and TEI values for each typology in each ECU, which could be useful for a further analysis in those geographical areas.

The third spreadsheet (*Intervencoast_tables_boxplot.xlsx*) includes the data filtered and organised to obtain the graphs 4, 5A and 5B of the article [1]. These calculations have a higher level of complexity than those of the second spreadsheets, because they include more robust statistical analysis. Initially, Fig. 4 of [1] is a box plot analysis based on the Tukey Test, which shows the TEI extreme and mild outliers in three filtered scenarios (29, 26 and 25 typologies). The next datasheet shows the data used for the graphs 5A and 5B of [1], which use the conditional format option of MS Excel to show graphically the value of TEI for each typology and ECU and the percentage of overall TEI.

**Fig. 4:**
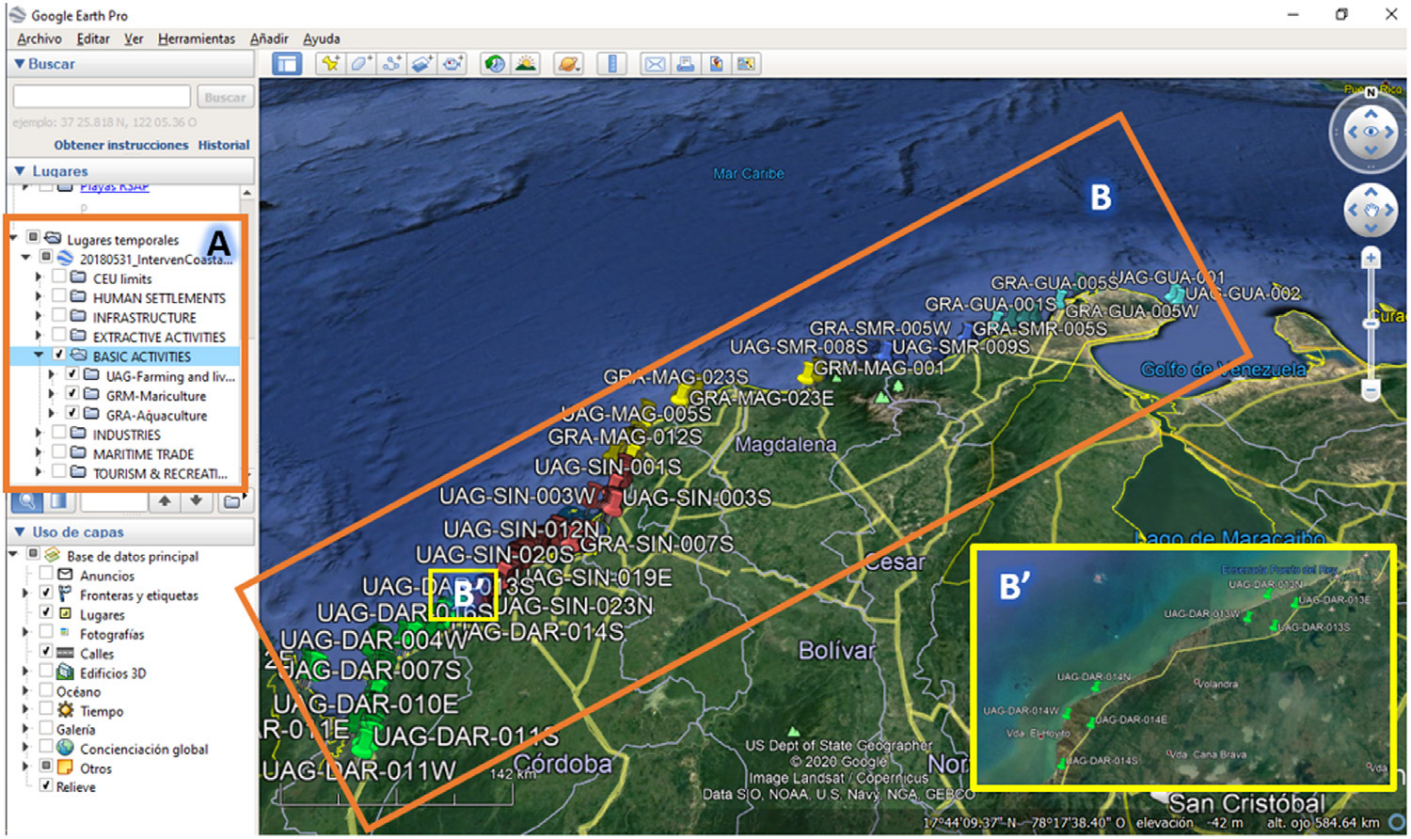
Examples of captures of kml files with the inventory of human interventions in the study area (A: categories of human interventions; B: Study area with all position marks (3957); B’: Zoom of smaller geographical area where the position marks are distinguishable).

The two Google Earth files (kmz) that complement the dataset show the geographical location of each position mark describing the human interventions in the study area, which comprise the complete inventory. Those two files have the same information, but organised in a different manner, in order to make easy their consultation and manipulation. One of the kmz files groups the 3957 position marks for the 38 typologies of human interventions. Meanwhile, another file groups the position marks within the five ECU. These two files are of the utmost importance for any researcher or practitioner interested to see some specific human intervention or geographical sector, because the software of Google Earth allows to navigate virtually on the study area (Fig. 4).

## 2 Experimental design, materials, and methods

### 2.1 Study area

**Fig. 5:**
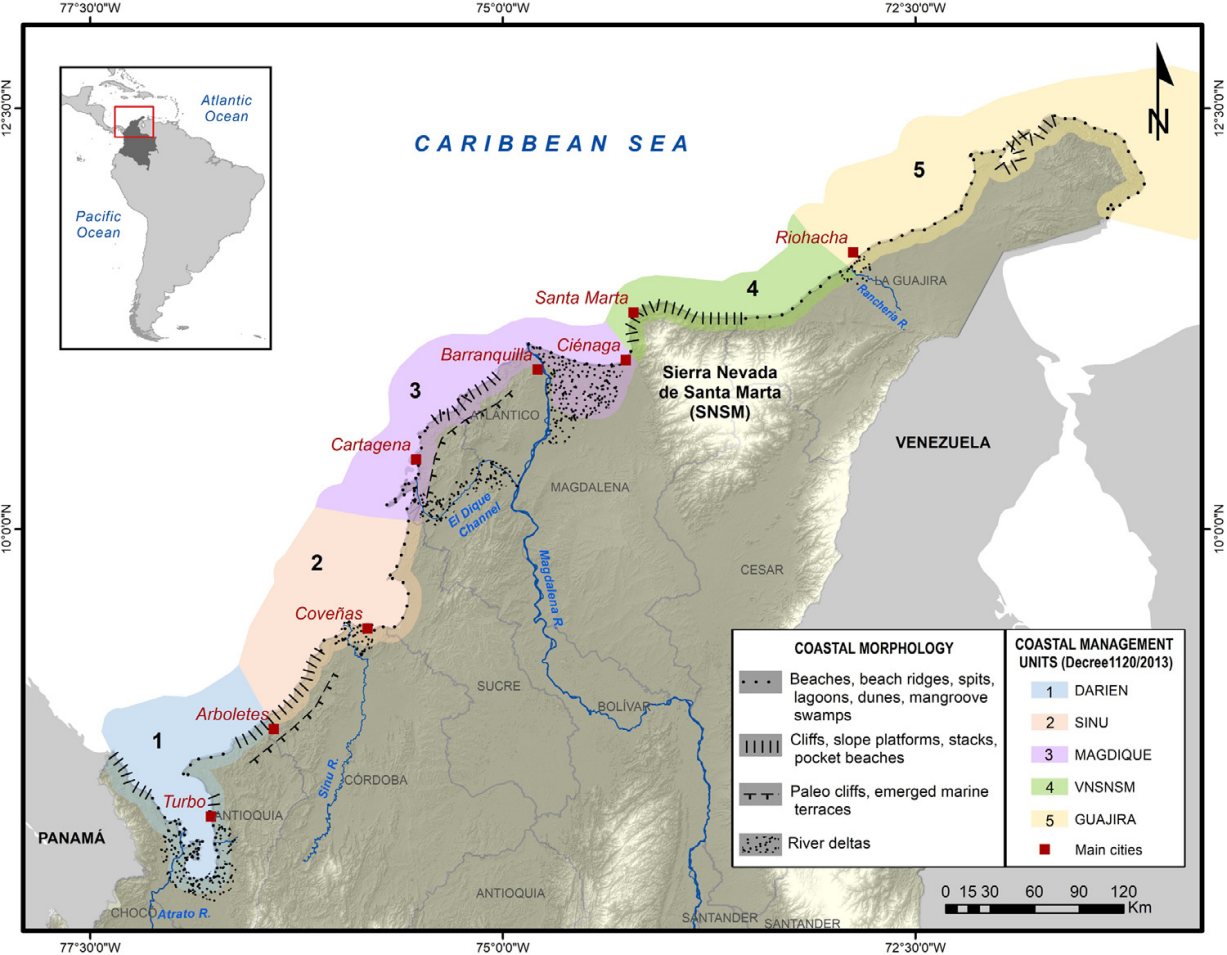
Study area: Caribbean coast of Colombia.

Colombia has officially three coastal zones, according to Decree 1120 of 2013: Continental Caribbean Coast, Insular Caribbean Coast and Pacific Coast. The dataset shown in this article covers the first of them. In the same Decree, five Environmental Coastal Units (ECU) are defined for the study area: La Guajira peninsula (GUAJIRA); the northern slope of the Sierra Nevada of Santa Marta (VNSMR); Magdalena Delta and Canal del Dique (MAGDIQUE); Sinu Delta (SINU); and Darien Gulf (DARIEN). Their boundaries are shown in Fig. 5.

The approximately 1700 km shoreline of the study area alternates between deltaic plains and low coasts with high coasts of mountainous segments [2]. The low-lying coasts contain beaches, sand barriers and spits, normally associated with lagoons and mangrove swamps. On the other hand, the high coast sectors are represented by cliffs of sedimentary rocks in the northernmost end (La Guajira) and the middle part (between Barranquilla and Cartagena city), while the cliffs around the *Sierra Nevada de Santa Marta* massif and the southernmost end (Panama border) correspond to more resistant igneous and metamorphic rocks [3]. Between the deltas of the Magdalena and Atrato rivers, the coast is backed by Holocene marine terraces and influenced by the mud diapiric phenomena [4]. This last one is a process reshaping the sea bottom trigged by the rising of low density material deforming the upper sediment layers or outflowing of the continental shelf; in both cases shoals and islands can form, such as El Rosario archipelago near Cartagena city [5]. Similar phenomena occur at the coast (e.g. mud volcanos of Totumo and Arboletes) producing tourist attractions, but also a relevant risk for the surrounding population.

According to National Statistics Institute [6], the Caribbean region of Colombia has large areas (departments of Choco, Cordoba, Sucre, Magdalena, La Guajira) with socioeconomic development based on the primary sector. The industries and the third economic sector is highly concentrated in the densest areas between Cartagena and Santa Marta, which represents less than a third part of the coastline. Furthermore, the most populated cities of the study area (Barranquilla, Cartagena, Santa Marta, Cienaga and Riohacha) represent one sixth of the most populated cities (over 3 million inhabitants) in the country, and still concentrates little over 6% of the total national population [6]. Related to the economic infrastructure, port activity is highly concentrated in Barranquilla and Cartagena, where the biggest port facilities are placed [7]. In addition, tourist activity within the ‘3S’ tourism category (Sun, Sea and Sand; [8]), is highly concentrated in Santa Marta, Cartagena, and Coveñas [6,9].

### 2.2 Inventory of human interventions

The inventory of human intervention in the study area was compiled using the structure of coastal uses and activities proposed by Botero [10]. This scheme served as a reference for selecting the 38 types of human interventions identified through Google Earth. A code system was defined to represent the type of intervention using an alphanumerical coding: the first three letters represent the ECU where the intervention is located, the following three letters represent the intervention typology, and the last three digits stands for the numerical order.

The instrumentation for data collection relied on the software Google Earth because it provides easy access to numerous satellite images of the study area with adequate horizontal and vertical resolution to observe the earth relief and identify geomorphological units, both natural and anthropogenic [11,12]. The image information was mostly sourced from the collection of satellite images of Google Earth, but alternative imagery services were also used (Nokia, Bing, ESRI). The majority of the georeferencing work was done through Google Earth; although, other geographic information systems, such as ArcMap from ESRI or the open source gvSIG, were used to assist the registration of the interventions within the alternative imagery inputs.

### 2.3 Simplified environmental impact assessment

The environmental impact assessment was calculated from a simplified version of the Conesa [13] equation. Initially, the frequency of human interventions by each typology was counted in the MS Excel datasheet, using the function “COUNTIFS” to extract the amount of interventions at a desired typology (FREQ). Later, the values for each attribute of environmental impact (EXT, INT, REV, PER) were allotted according to the levels defined by Conesa [13]. Stemming from these values, the UEI was calculated with the MS Excel function “SUM” divided by the maximum environmental impact value (32). Finally, the TEI value was calculated multiplying the UEI score with the frequency of occurrence previously counted. Details about interpretation and the pertinence of each parameter and calculation are in [1].

## Declaration of Competing Interest

The authors declare that they have no known competing financial interests or personal relationships that might have appeared to influence the work reported in this paper.

## Appendix Supplementary materials

Image, application 1

Image, application 2

Image, application 3

Image, application 4
